# An inflammation‐associated ferroptosis signature optimizes the diagnosis, prognosis evaluation and immunotherapy options in hepatocellular carcinoma

**DOI:** 10.1111/jcmm.17780

**Published:** 2023-05-30

**Authors:** Wan‐Yuan Ruan, Lu Zhang, Shan Lei, Zhi‐Rui Zeng, Yu‐Shi Yang, Wen‐Peng Cao, Qiu‐Yao Hao, Min Lu, Xiao‐Bin Tian, Pai‐Lan Peng

**Affiliations:** ^1^ Department of Gastroenterology The Affiliated Hospital of Guizhou Medical University Guiyang China; ^2^ School of Clinical Medicine Guizhou Medical University Guiyang China; ^3^ School of Basic Medicine Guizhou Medical University Guiyang China; ^4^ Department of Pathology The Affiliated Hospital of Guizhou Medical University Guiyang China; ^5^ Department of Gastroenterology, Zhujiang Hospital Southern Medical University Guangzhou China

**Keywords:** diagnosis, hepatocellular carcinoma, immunotherapy optimisation, inflammation‐associated ferroptosis gene, prognosis evaluation

## Abstract

Inflammation and ferroptosis crosstalk complexly with immune microenvironment of hepatocellular carcinoma (HCC), thus affecting the efficacy of immunotherapy. Herein, our aim was to identify the inflammation‐associated ferroptosis (IAF) biomarkers for contributing HCC. A total of 224 intersecting DEGs identified from different inflammation‐ and ferroptosis‐subtypes were set as IAF genes. Seven of them including *ADH4*, *APOA5*, *CFHR3*, *CXCL8*, *FTCD*, *G6PD* and *PON1* were used for construction of a risk model which classified HCC patients into two groups (high and low risk). HCC patients in the high‐risk group exhibited shorter survival rate and higher immune score, and were predicted to have higher respond rate in immune checkpoint inhibition (ICI) therapy. Levels of the seven genes were significantly changed in HCC tissues in comparison to adjacent tissues. After inserting the gene expression into the risk model, we found that the risk model exhibited the higher diagnostic value for distinguish HCC tissues compared each single gene. Furthermore, HCC tissues from our research group with high‐risk score exhibited more cases of microsatellite instability (MSI), heavier tumour mutational burden (TMB), higher expression level of PDL1 and cells with CD8. Knockdown of *APOA5* reduced HCC cell proliferation combining with elevating inflammation and ferroptosis levels. In conclusion, we considered *APOA5* maybe a novel target for suppressing HCC via simultaneously elevating inflammation and ferroptosis levels, and signature constructed by seven IAF genes including *ADH4*, *APOA5*, *CFHR3*, *CXCL8*, *FTCD*, *G6PD* and *PON1* can act as a biomarker for optimising the diagnosis, prognosis evaluation and immunotherapy options in HCC patients.

## 1 INTRODUCTION

Hepatocellular carcinoma (HCC) ranks sixth in morbidity and third in mortality of tumour‐related deaths worldwide.^1^ Surgical resection is the main therapeutic strategy for early‐stage HCC. However, most of the individuals are diagnosed at an advanced stage, thus losing the opportunity for surgical treatment.^2^ Moreover, HCC cells have a high resistance to conventional cytotoxic chemotherapeutic agents, limiting the benefit of chemotherapy in patients with HCC.^3^ Since 2008, several tyrosine kinase inhibitors, including sorafenib and regorafenib, have been approved for first‐ and second‐line treatments of HCC, whereas the overall survival rate still has no improvement.^4^ In recent years, approval of immunotherapy, especially immune checkpoint inhibitor (ICI) therapy, has greatly relieved this embarrassing treatment status, and significantly improves the outcome of part of individuals. Unfortunately, majority of HCC individuals are defeated to respond to ICI.^5^ In patients who fail respond to ICI, immune‐related adverse reactions including dermatotoxicity, endocrine system toxicity, pneumonia and gastrointestinal toxicity turn into greater challenge during therapy.^6^ Therefore, exploration of novel biomarkers for immunotherapy optimisation in HCC individuals was urgent.

Inflammation is a hallmark of cancer, and its link with the progression of cancer is well established. In fact, inflammation plays a double‐edged role in tumours.^7^ Studies have shown that transient inflammation exhibit anti‐tumour effects, while chronic persistent inflammation can affect the plasticity of tumour cells by regulating cell differentiation, immune cell polarisation in microenvironment and other processes, hence facilitating tumour proliferation, metastasis and drug resistance.^8^ Similarly, the role of inflammation in immune regulation is complex. Immunosuppressive inflammatory microenvironment is commonly formed in tumour tissues; therefore, tumour cells often escape from the killing of immune cells including T cells.^9^ However, during immune checkpoint inhibition (ICI) therapy, the tumour microenvironment is remodelled, and tumour‐promoting inflammation is transformed into tumour‐suppressive inflammation.^10^

Ferroptosis is a specific cell death model that depends on lipid peroxidation process, and it was different from procedural death in morphology and molecular mechanism.^11^ Clinical researchers found that ferroptosis commonly exist in cancer cells while patients obtain radiotherapy, chemotherapy and tumour immunotherapy, indicating that ferroptosis activation is the strategy to inhibit cancer.^12^ Ferroptosis has crosstalk with inflammation and immunotherapy. Previous studies indicated that lipid peroxides produced during ferroptosis are identifying signals to accelerate recognition, phagocytosis and processing of tumour antigens by dendritic cells, thus intentinonally activating cytotoxic T lymphocytes to improve the efficacy of tumour immunotherapy.^13^ Inflammation is an important factor involve in the activation of ferroptosis, and this phenomenon can be observed in the tissues after ICI therapy.^14^ Therefore, inflammation induced the activation of ferroptosis during ICI therapy is generally beneficial to anti‐tumour, and can act as biomarker to reflect the response of ICI.

Herein, our aim was to identify inflammation‐associated ferroptosis (IAF) biomarkers for diagnosis and therapy of HCC. We demonstrated that the signature constructed by seven IAF genes including *ADH4*, *APOA5*, *CFHR3*, *CXCL8*, *FTCD*, *G6PD* and *PON1* can act as a biomarker for optimising the diagnosis, prognosis evaluation and immunotherapy options in patients with HCC. Among the IAF genes, *APOA5* was a novel target for suppressing HCC via simultaneously elevating inflammation and ferroptosis levels.

## 2 MATERIALS AND METHODS

### 2.1 Data acquisition and pretreatment

Train cohort containing the RNA‐sequencing profile of 369 patients with HCC and their clinical characters information was downloaded from The Cancer Genome Atlas (TCGA; https://portal.gdc.cancer.gov/). Test cohort containing the RNA‐sequencing information and clinical characters of 237 HCC patients was gained in International Cancer Genome Consortium (ICGC; https://dcc.icgc.org/). Prior to analysis, genes with missing value >50% were removed, and then gene expression profile was performed to normalize.

### 2.2 Subtypes cluster

Inflammation‐related genes were referred to the research of Danaher et al.,^15^ while ferroptosis‐related genes were referred to the terms of ‘WP_FERROPTOSIS’ in Gene Set Enrichment Analysis (GSEA, http://www.gsea‐msigdb.org/gsea/index.jsp). Then, the inflammation‐ and ferroptosis‐associated genes were used to conduct cluster analysis by the R package ‘ConsensusClusterPlus’, respectively. The cluster number was set as 3. Then, the difference of the overall survival (OS) between the subtypes was determined by Kaplan–Meier method. *p* < 0.05 was considered different.

### 2.3 Identification of differentially expressed genes (DEGs)

The DEGs between the different subtypes of HCC were analysed by the limma package (Version: 3.1.6). Genes with the |Log2 foldchange (LogFC)| ≥ 1 and adjust *p* value <0.05 were DEGs. Intersecting DEGs between inflammation‐ and ferroptosis‐subtypes were set as IAF genes, and used for further analysis.

### 2.4 Enrichment analysis of IAF genes

KEGG and GO analysis of IAF genes were analysed in the DAVID Bioinformatics Resources (https://david.ncifcrf.gov/; version: v2022q4). Enrichment GO terms contained biological process (BP) and molecular function (MF) terms. Enrichment terms with *p* < 0.05 were visualized.

### 2.5 Risk model establish based on significant IAF genes

In order to construct a risk model by IAF genes, we first performed LASSO analysis to remove similarity factors and reduce the scope by adding penalty parameter using the R package ‘glmnet’ (Version: 4.1‐6). Then, the effects on the OS of HCC patients of residual IAF genes was detected by performing univariate and multivariate regression analysis. By multiplying levels of genes and their corresponding regression coefficients deduced from multivariate Cox regression analysis, risk model was constructed. The HCC tissues were then divided into high‐ and low‐risk groups followed by calculating the risk score of each tissue. The OS difference between the two groups was analysed by Kaplan–Meier method, while the effects of risk score on OS of HCC patients were analysed by univariate and multivariate regression analysis. *p* < 0.05 was cut‐off.

### 2.6 Immune‐related analysis

ESTIMATE (https://bioinformatics.mdanderson.org/estimate/; version: 1.0.11) was utilized to measure the immune signatures of HCC tissues, including immune, stromal and ESTIMATE score. For immune cell infiltration analysis, built‐in data LM22 containing gene expression characteristics of 22 immune cells was set as reference. Cibersort package (version: 1.04) was conducted to measure the levels of 22 immune cells in HCC tissues via matching the reference data. The *t*‐test was performed to analysed the differences via the cut‐off as *p* < 0.05.

### 2.7 Tumour immune dysfunction and exclusion (TIDE) analysis

For prediction of the respond rate of HCC tissues in the two groups (high and low risk) after ICI treatment, TIDE database (http://tide.dfci.harvard.edu/) was conducted. The *t*‐test was performed to analysed the differences of dysregulation, exclusion and TIDE score between the two groups, while difference of respond ratio was determined via chi‐squared test. *p* < 0.05 was cut‐off.

### 2.8 Tissues collection

Total 40 pair HCC tissues and corresponding adjacent tissues were collected from Affiliated Hospital of Guizhou Medical University with the approve of the Human Ethics Committee of Guizhou Medical University (Approval number: 2022‐282). All individuals had obtaining written informed consent. All of them had not receive chemotherapy, target therapy and immunotherapy prior to tissues collection. Similarly, all patients had complete medical records including the information of microsatellite instability (MSI) and tumour mutational burden (TMB). MSI was determined by detecting the mutation of NR‐21, BAT‐26, NR‐24, BAT‐25 and MONO‐27 site. While HCC patients with mutation site = 0, =1 or ≥2, was set as microsatellite stability (MSS), MSI‐low (MSI‐L) and MSI‐high (MSI‐H), respectively. All tissue specimen was collected in −80°C prior to perform experiments.

### 2.9 RT‐qPCR experiments

Total RNA in HCC tissues and corresponding adjacent tissues were obtained by TRIZOL reagent (Solarbio, Beijing, China). Then, total 800 ng mRNA was used to synthesize first‐strand cDNA utilising RT Master Mix for qPCR II (gDNA digester plus; MCE). Fluorescence amplification quantification was performed using SYBR green reagent (MCE). Relative expression of target genes was referred to glyceraldehyde‐3‐phosphate dehydrogenase (*GAPDH*). Primers used in fluorescence amplification quantification was shown as follows: alcohol dehydrogenase 4 (*ADH4*) forward primer 5′‐ AGTTCGCATTCAGATCATTGCT ‐3′, *ADH4* reverse primer 5′‐ CTGGCCCAATACTTTCCACAA ‐3′; apolipoprotein A5 (*APOA5*) forward primer 5′‐ GCTGGTGGGCTGGAATTTG ‐3′, *APOA5* reverse primer 5′‐ CTCGGCGTATGGGTGGAAG‐3′; complement factor H‐related 3 (*CFHR3*) forward primer 5′‐ TGCTAATGGACAAGTGAAACCTT ‐3′, *CFHR3* reverse primer 5′‐ GGCAACTTCTGTAGAGTTACCC‐3′; C‐X‐C motif chemokine ligand 8 (*CXCL8*) forward primer 5′‐ TTTTGCCAAGGAGTGCTAAAGA ‐3′, *CXCL8* reverse primer 5′‐ AACCCTCTGCACCCAGTTTTC ‐3′; formimidoyltransferase cyclodeaminase (*FTCD*) forward primer 5′‐ GGAATGCGTCCCCAACTTTTC ‐3′, *FTCD* reverse primer 5′‐ TGTCGATAAGTCGGGAAGCTAC ‐3′; glucose‐6‐phosphate dehydrogenase (*G6PD*) forward primer 5′‐ CGAGGCCGTCACCAAGAAC ‐3′, *G6PD* reverse primer 5′‐ GTAGTGGTCGATGCGGTAGA −3′; paraoxonase 1 (*PON1*) forward primer 5′‐ CTGATTGCGCTCACCCTCTT ‐3′, *PON1* reverse primer 5′‐ CGGAGAGCATTAAGTCGTGTTTG ‐3′; *GAPDH* forward primer 5′‐ GGAGCGAGATCCCTCCAAAAT ‐3′, *GAPDH* reverse primer 5′‐ GGCTGTTGTCATACTTCTCATGG −3′.

### 2.10 Immumohistochemical staining

Total 40 HCC tissues were cut into 2 μm‐thick paraffin specimens. The specimens were conducted antigen‐repair in citrate (Beyotime) followed by dewaxing. The tissues were then infiltrated in 3% H_2_O_2_ for 10 min and 8% bovine serum albumin for 15 min for prevention of non‐specific binding. Diluted anti‐PDL1 antibody (1:50,00; Cat No. 66248‐1‐Ig; Proteintech) and anti‐CD8 antibody (1:10,000; Cat No. 66868‐1‐Ig; Proteintech) were incubated with the sections overnight. Sections were washed by PBS for twice, and second antibodies were incubated with sections for 2 h. DAB solution (Solarbio) and haematoxylin (Solarbio) were used to stain the sections for 30 and 10 s, respectively. Finally, the HCC sections after treatment were dehydrated, transparent and sealed.

### 2.11 Cell culture and transfection of small interfering RNAs (siRNAs)

HCC cell HepG2 and Huh7 were obtained from ATCC database, and cultured in DMEM medium (Hyclone) with 10% FBS (Hyclone) at 37°C environment. Targeting APOA5 siRNAs and scramble siRNA were constructed by iGeneBio. The sequences of siRNAs for targeting APOA5 was 5′‐ GAGCAAGACCUCAACAAUAUG −3′ and 5′‐ CGAUGGAUCUGAUGGAGCAGG −3′. The transfection of siRNAs were conducted by lipo2000 (Invitrogen) according to process provided by manufacturer.

### 2.12 Western blotting

Total protein in HCC cells were extracted by RIPA buffer (Boster) containing with 1% PMSF (Boster). Following by detecting concentration by BCA method, proteins were separated by 12.5% SDS‐PAGE, and transfected onto PVDF membranes (Invitrogen). After blocking with TBST containing 5% milk powder, anti‐APOA5 (1:500; cat no. A1424; Abconal) and anti‐β‐actin (1:10,000; cat no. AC026; Abconal) were added overnight at 4°C. After washing by secondary antibodies and visualising by ECL reagent (Invitrogen), relative expression of APOA5 was calculated via normalising to β‐actin.

### 2.13 Enzyme‐linked Immunosorbent assay (ELISA) experiment

Total 1 × 10^5^ negative control (NC) HepG2 and Huh7 cells and cells with APOA5 knockdown were set into per well for 24 h and then lysed. The TNF‐α and IFN‐γ in cell lysate of per sample was determined with corresponding ELISA kit (Beijing Tiantan Biological Products co., LTD). The relative levels of TNF‐α and IFN‐γ were normalized to protein concentration.

### 2.14 Relative experiments for detecting ferroptosis

Based on manufacturer's instructions, Glutathione Assay Kits (Invitrogen) were utilized to analyse the levels of glutathione (GSH) in HepG2 and Huh7 cells; Lipid Peroxidation (MDA) Assay Kit (Invitrogen) was used to detect the levels of MDA in HepG2 and Huh7 cells; while the iron concentrations were analysed using an Iron Assay Kit (Invitrogen).

### 2.15 Reactive oxygen (ROS) detection

The cells were seeded in 6‐well plates in triplicate and cultured for the appropriate period of time until 70% confluence was reached. To measure ROS levels in whole cells, the cells were cultured for 24 h in fresh DMEM and stained with 10 μM 2′,7′‐dichlorodihydrofluorescein diacetate (DCFH‐DA; Invitrogen, USA). The ROS level was assessed using flow cytometry and analysed using FlowJo (version: 7.4.1).

### 2.16 Cell prolfieration and colony formation detection

For CCK‐8 assays, HepG2 and Huh7 cells were seeded in 96‐well plates in triplicate with the density of 3 × 10^3^ per well. After culturing for 48 h, 10 μL CCK‐8 was added in per well for 2 h. Finally, the absorbancy of each well was detected in 450 nm in microplate reader. For colony formation assays, HepG2 and Huh7 cells were seeded in 6‐well plates in triplicate with the density of 2 × 10^3^ per well. After 14 days, culture medium was removed, while cell colony was fixed with paraformaldehyde and fixed with crystal violet. The condition of cell colony in per well was photographed and calculated.

### 2.17 Data statistics

The data results were analysed in SPSS 19.0. Differences between the two groups were determined by t‐test, while one‐way analysis of variance was used to measure the differences between multigroups. *p* < 0.05 was significant.

### 2.18 Inflammation‐ and ferroptosis‐ molecular subtypes was associated with HCC patient prognosis

**FIGURE 1:**
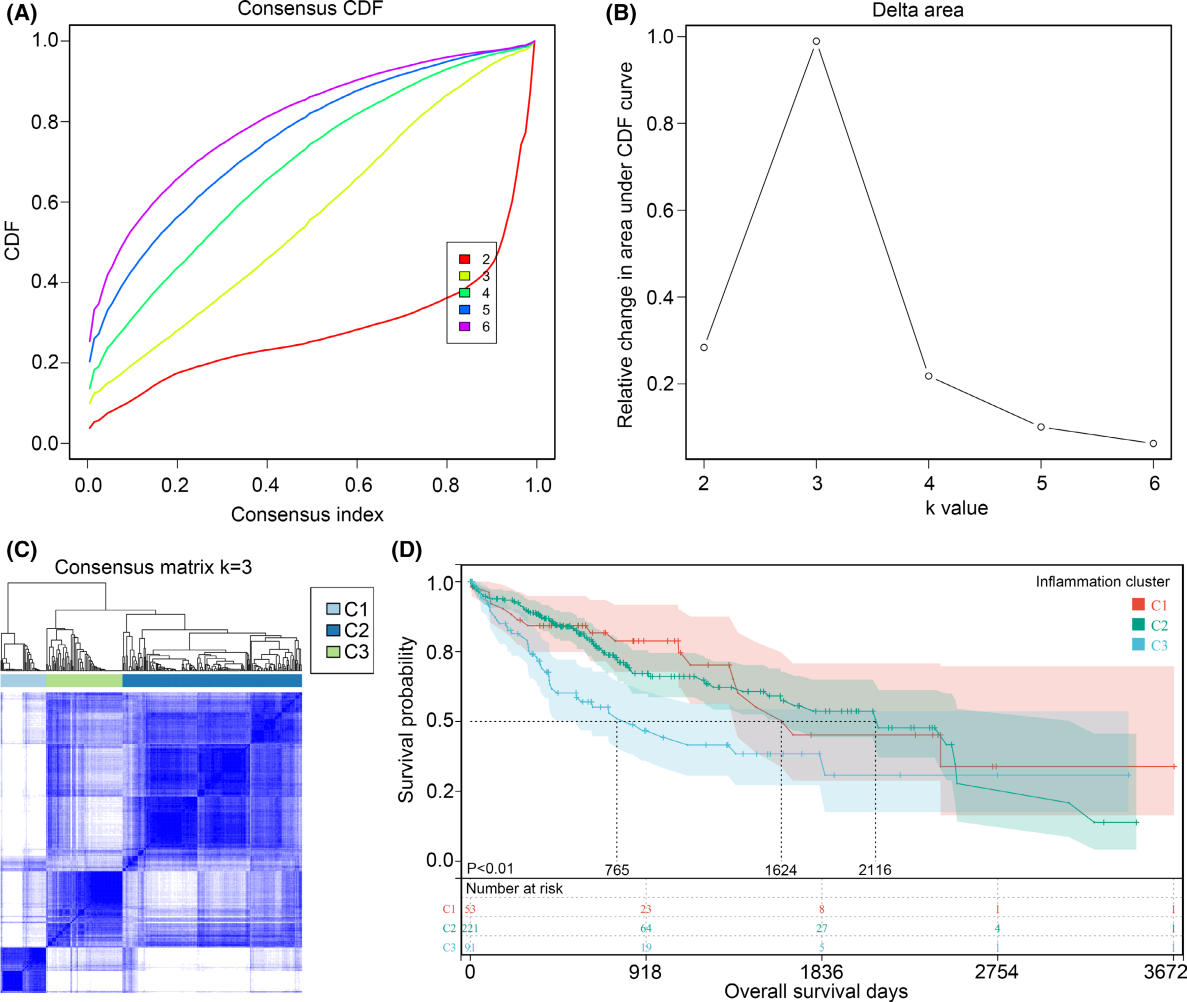
Cluster of HCC samples based on inflammation‐related genes. (A) Consensus clustering CDF with *k* valued 2–6. (B) Relative change in area under CDF curve for *k* = 3. (C) Consensus clustering matrix when *k* = 3. (D) The overall survival rate of C1, C2, C3 inflammation‐related cluster.

**FIGURE 2:**
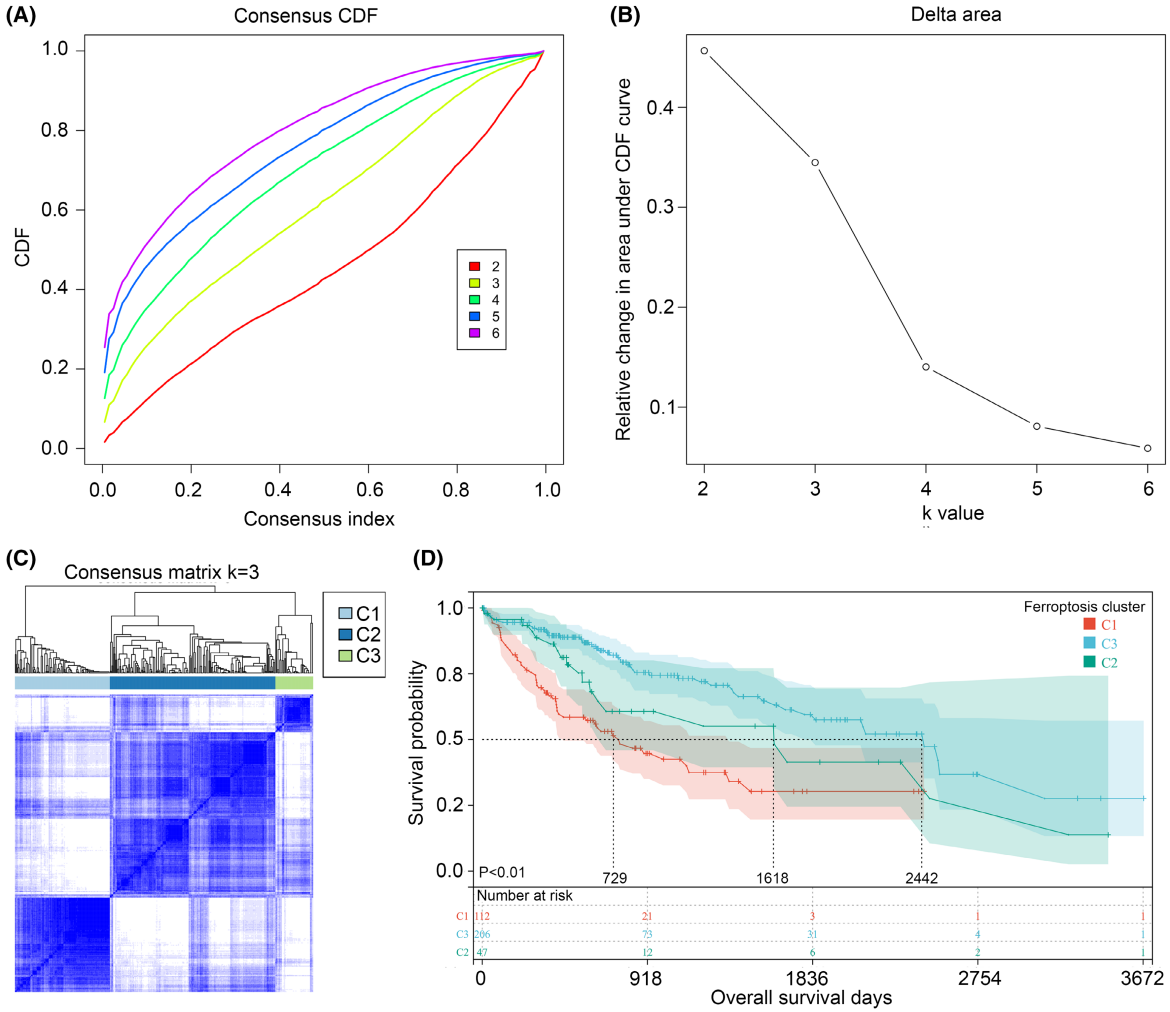
Cluster of HCC samples based on ferroptosis‐related genes. (A) Consensus clustering CDF with *k* valued 2–6. (B) Relative change in area under CDF curve for *k* = 3. (C) Consensus clustering matrix when *k* = 3. (D) The overall survival rate of C1, C2, C3 ferroptosis‐related cluster.

In order to obtain different subtype HCC tissues with inflammation signature, characteristic inflammation‐related genes were conducted for consensus clustering analysis (Figure 1A,B). Total three subtypes (C1, C2 and C3 inflammation cluster) of HCC with different inflammation signature were obtained (Figure 1C). Kaplan–Meier method suggested that, compared with the OS of HCC patients in C1 and C2 inflammation cluster, HCC patients in C3 inflammation cluster had the shortest OS time (Figure 1D; HR = 1.55, 95% CI = 1.17–2.05). Similarly, characteristic ferroptosis‐related genes enrolled in consensus clustering analysis (Figure 2A,B), and three subtypes (C1, C2 and C3 ferroptosis cluster) of HCC with different ferroptosis signature were gained (Figure 2C). Compared with the OS of HCC patients in C2 and C3 ferroptosis cluster, HCC patients in C1 ferroptosis cluster had the shortest OS time (Figure 2D; HR = 1.75, 95% CI = 1.30–2.33). These evidences indicated that inflammation‐ and ferroptosis‐ molecular subtypes was associated with HCC patient prognosis.

### 2.19 Identification of IAF genes in HCC

**FIGURE 3:**
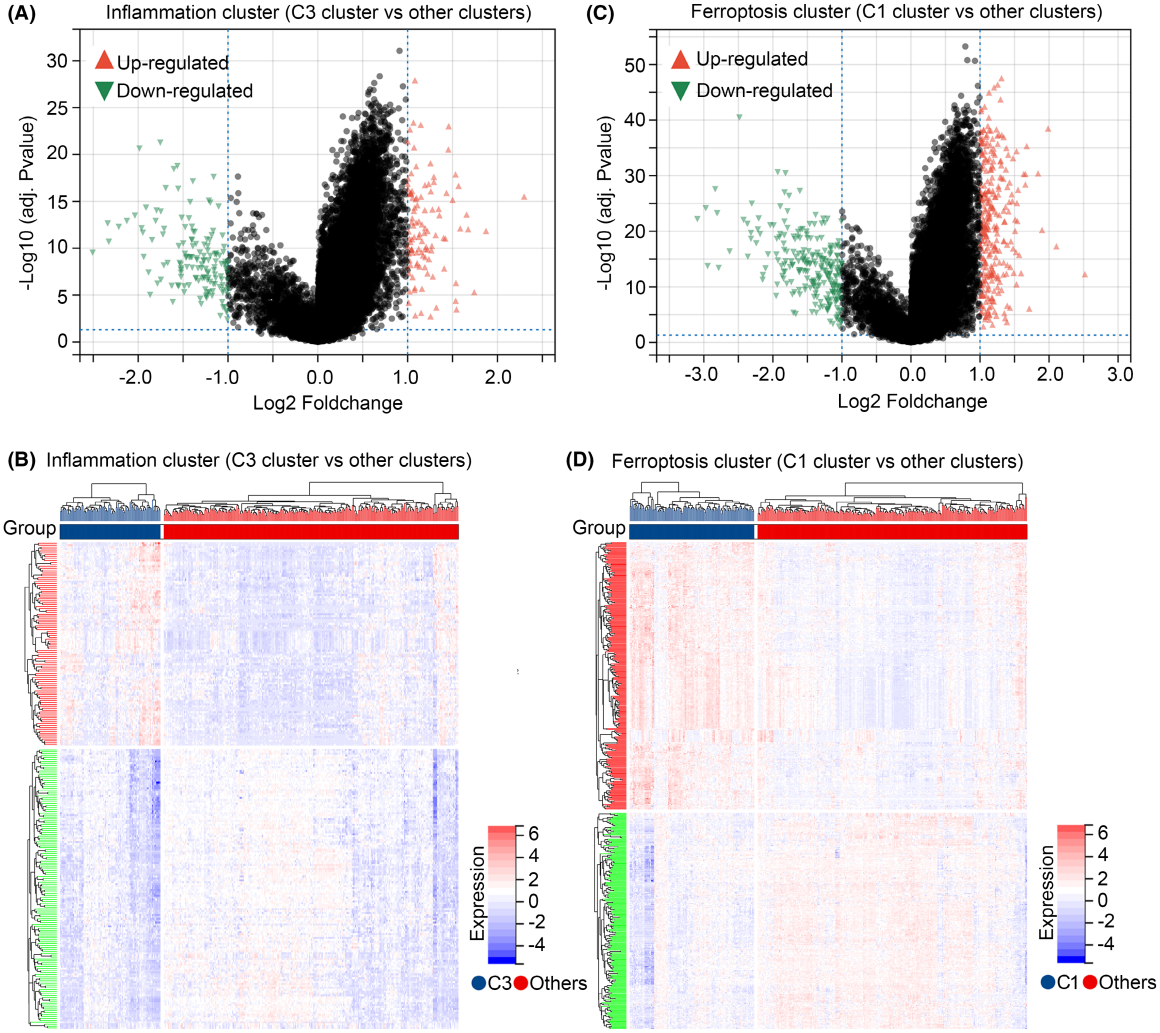
Identification of differentially expressed genes (DEGs) between inflammation‐ and ferroptosis‐related clusters. (A, B) DEGs between C3 inflammation cluster and other inflammation clusters. (C, D) DEGs between C1 ferroptosis cluster and other ferroptosis clusters.

**FIGURE 4:**
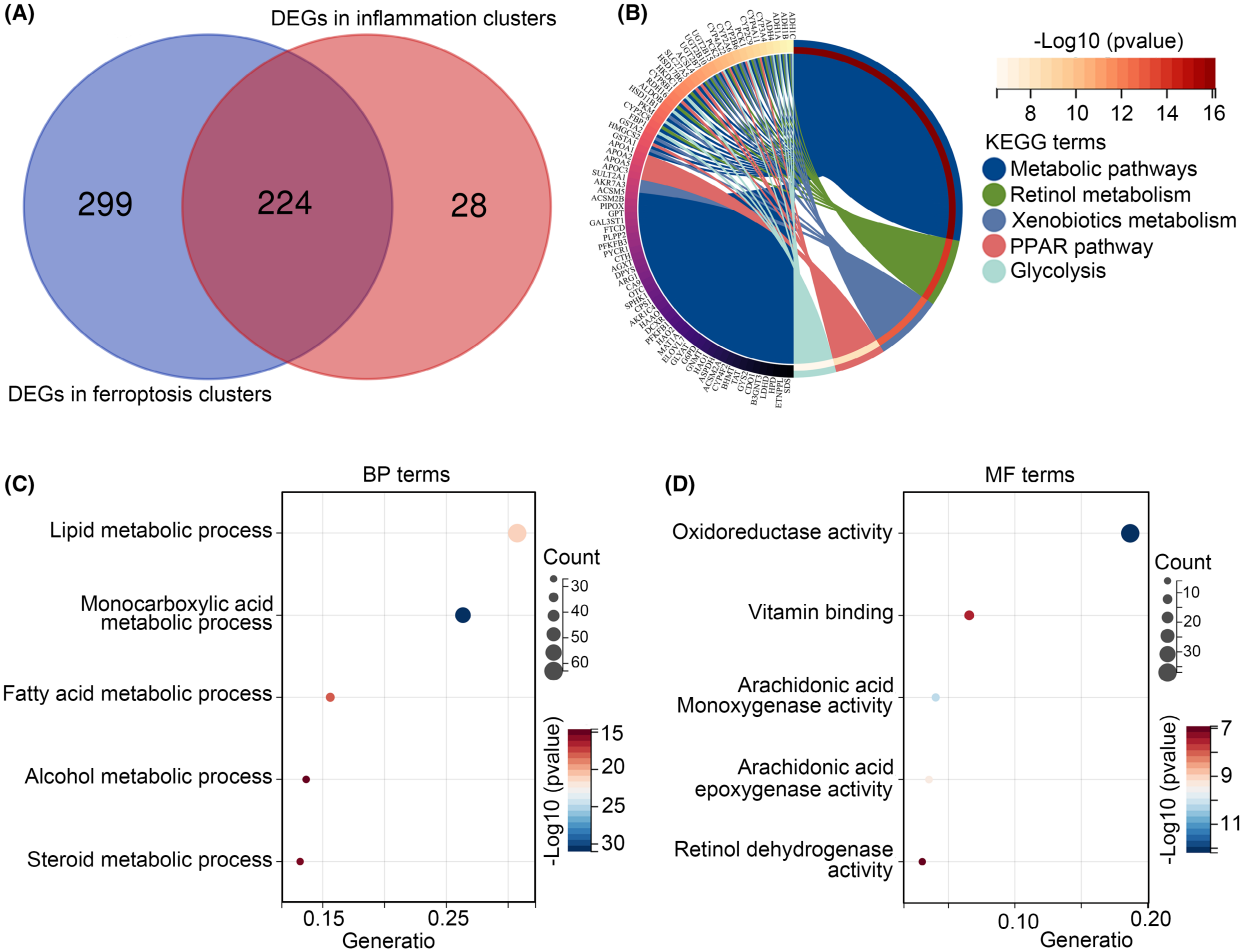
Analysis of intersecting DEGs identified from different inflammation‐ and ferroptosis‐subtypes. (A) Intersecting DEGs identified from different inflammation‐ and ferroptosis‐subtypes, and set as inflammation‐associated ferroptosis (IAF) genes. (B) KEGG terms of IAF genes enriched in. (C) Biological process (BP) terms of IAF genes enriched in. (D) Molecular function (MF) terms of IAF genes enriched in.

We performed DEGs analysis in inflammation clusters (C3 vs. others clusters), total 106 upregulated genes and 146 downregulated genes were found (Figure 3A,B; Supplemental Table S1). Similarly, through performing DEGs analysis in ferroptosis clusters (C1 vs. others clusters), total 289 upregulated genes and 234 downregulated genes were found (Figure 3C,D; Table S2). A total of 224 intersecting DEGs were found (Figure 4A) and set as IAF genes in HCC. KEGG analysis indicated that enrich terms of IAF genes were ‘metabolic pathways’, ‘retinol metabolism’, ‘xenobiotics metabolism’, ‘PPAR pathway’ and ‘glycolysis’ (Figure 4B). GO analysis exhibited that BP terms of IAF genes were enriched in ‘lipid metabolic process’, ‘monocarboxylic acid metabolic process’, ‘fatty acid metabolic process’, ‘alcohol metabolic process’ and ‘steroid metabolic process’ (Figure 4C), while MF terms of IAF genes were enriched in ‘oxidoreductase activity’, ‘vitamin binding’, ‘arachidonic acid monooxygenase activity’, ‘arachidonic acid epoxygenase activity’ and ‘retinol dehydrogenase activity’ (Figure 4D).

### 2.20 Construction of risk model based on significant IAF genes

**FIGURE 5:**
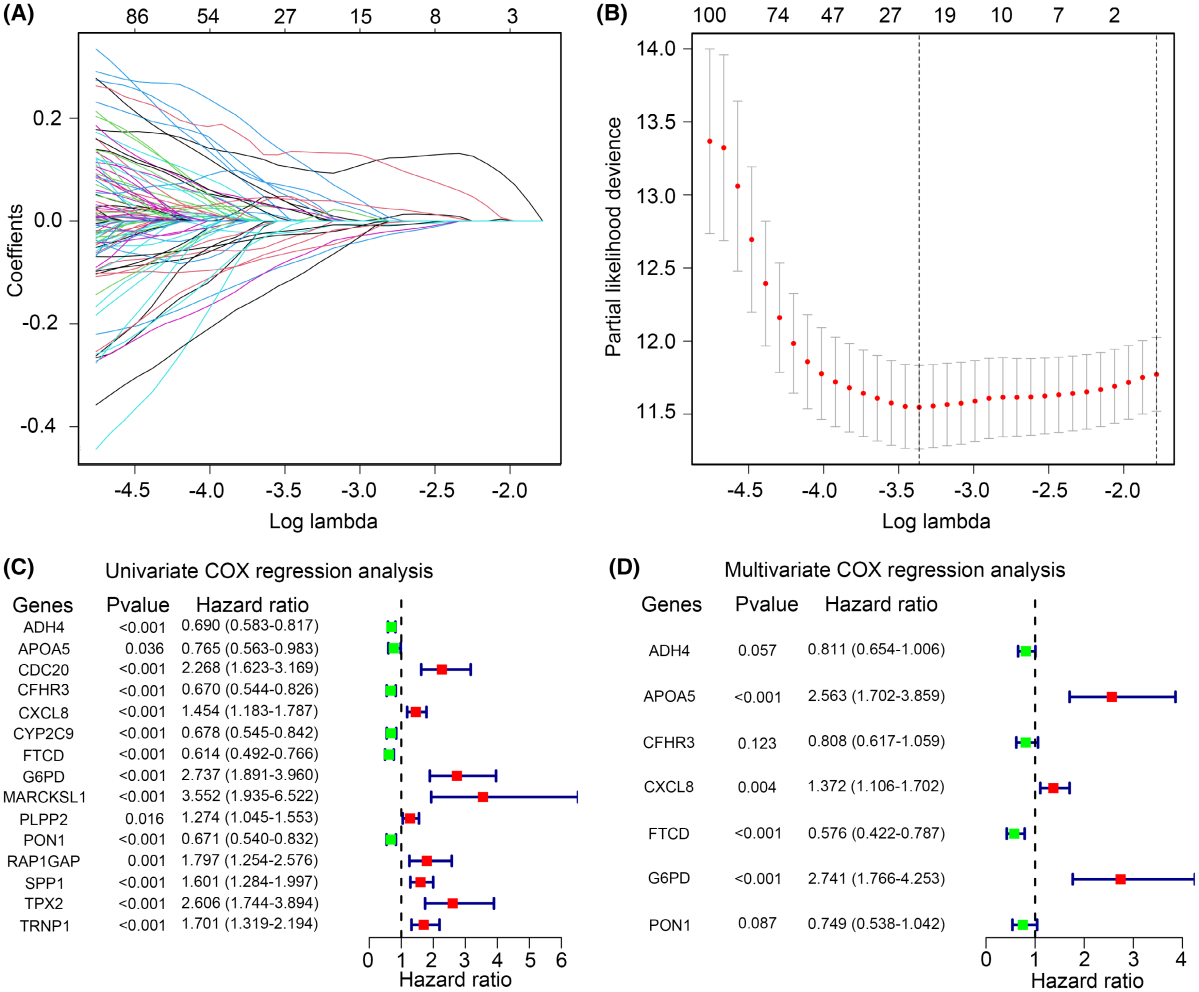
Construction of risk model based on inflammation‐associated ferroptosis (IAF) genes. (A, B) Lasso Cox analysis for IAF genes, total 23 IAF genes were retained. (C) Univariate Cox regression analysis for the 23 IAF genes. (D) Multivariate Cox regression analysis for the 23 IAF genes.

Total 224 IAF genes were enrolled in LASSO Cox analysis, and 201 genes with high similarity were removed (Figure 5A,B). Then, residual 23 genes were conducted for univariate Cox regression analysis. *ADH4* (HR = 0.690), *APOA5* (HR = 0.765), *CDC20* (HR = 2.268), *CFHR3* (HR = 0.670), *CXCL8* (HR = 1.454), *CYP2C9* (HR = 0.678), *FTCD* (HR = 0.614), *G6PD* (HR = 2.737), *MARCKSL1* (HR = 3.552), *PLPP2* (HR = 1.274), *PON1* (HR = 0.671), *RAP1GAP* (HR = 1.797), *SPP1* (HR = 1.601), *TPX2* (HR = 2.606) and *TRNP1* (HR = 1.701) was found to correlate with the OS of HCC patients (Figure 5C). These genes were used for multivariate Cox regression analysis, while *ADH4*, *APOA5*, *CFHR3*, *CXCL8*, *FTCD*, *G6PD* and *PON1* were conducted for construction of a risk model (Figure 5D). The risk model was risk score = −0.209 × *ADH4* + 0.941 × *APOA5* – 0.213 × *CFHR3* + 0.316 × *CXCL8*–0.551 × *FTCD* + 1.008 × *G6PD* – 0.289 × *PON1*.

### 2.21 Risk model served as prognostic biomarker in HCC individuals

**FIGURE 6:**
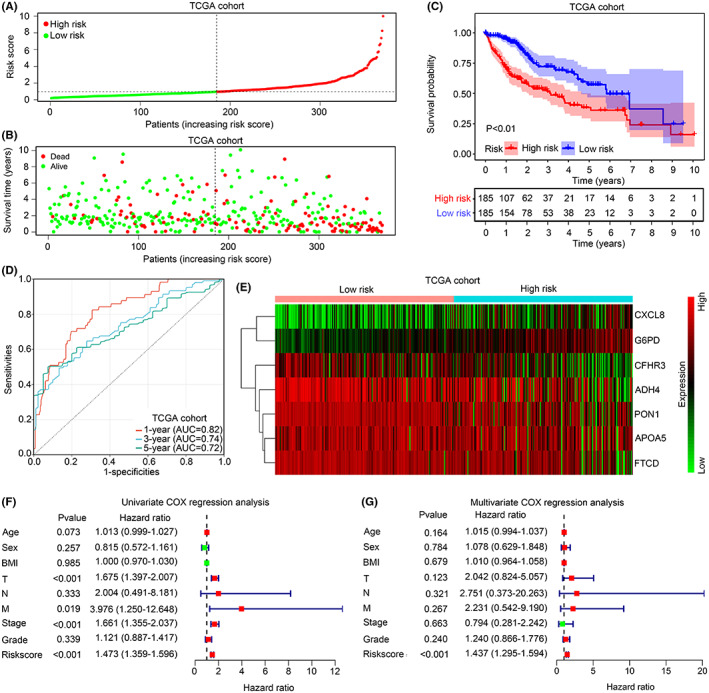
Risk model can act as prognostic biomarker for HCC tissues in TCGA. (A) HCC patients in TCGA were divided into high‐ and low‐risk group according to medium of risk score. (B) Higher death rate of HCC patients in the high‐risk group in TCGA. (C) Shorter overall survival rate in HCC patients in the high‐risk group in TCGA. (D) Prediction rate of risk model on the 1‐, 3‐ and 5‐year survival of HCC patients in TCGA. (E) Expression of *ADH4*, *APOA5*, *CFHR3*, *CXCL8*, *FTCD*, *G6PD* and *PON1* in HCC tissues between the high‐ and low‐risk groups in TCGA. (F, G) Univariate Cox regression analysis and multivariate Cox regression analysis for detecting the prognostic value of risk model in HCC patient in TCGA.

**FIGURE 7:**
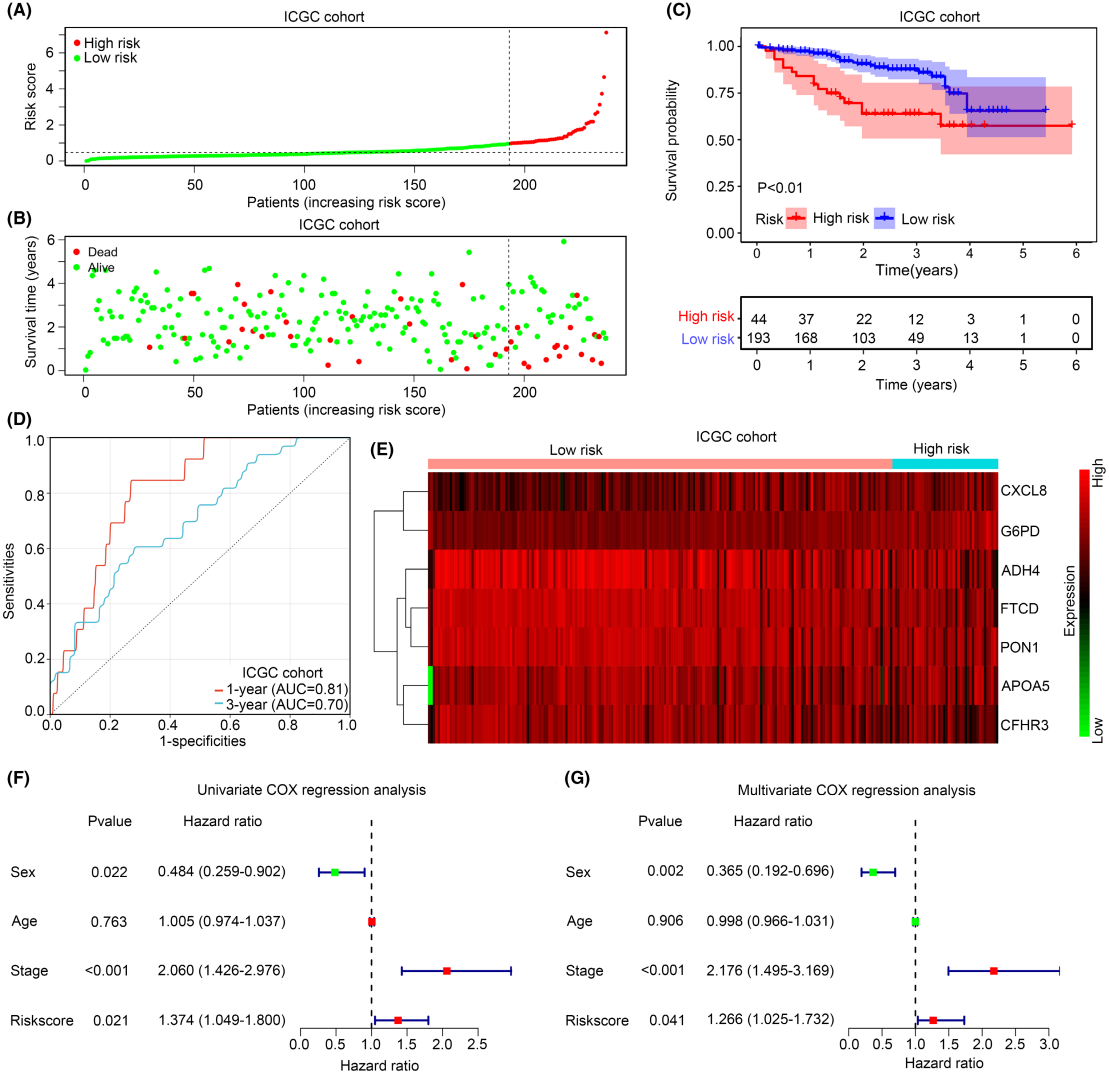
Risk model can act as prognostic biomarker for HCC tissues in ICGC. (A) HCC patients in ICGC were divided into high‐ and low‐risk groups according to medium of risk score. (B) Higher death rate of HCC patients in the high‐risk group in ICGC. (C) Shorter overall survival rate in HCC patients in the high‐risk group in ICGC. (D) Prediction rate of risk model on the 1‐year and 3‐year survival of HCC patients in ICGC. (E) Expression of *ADH4*, *APOA5*, *CFHR3*, *CXCL8*, *FTCD*, *G6PD* and *PON1* in HCC tissues between the high‐ and low‐risk groups in ICGC. (F, G) Univariate Cox regression analysis and multivariate Cox regression analysis for detecting the prognostic value of risk model in HCC patient in ICGC.

We checked the effects of risk model on prognosis evaluation in HCC tissues from the train cohort TCGA first. The HCC tissues in TCGA was divided into high‐ and low‐risk groups based on the median of risk score (Figure 6A). A higher proportion of deaths was found in the HCC individuals with high risk (Figure 6B). Compared the OS time in the low‐risk group, the OS time in HCC individuals with high risk were shorter (Figure 6C; HR = 2.27, 95% CI = 1.61–3.03). The prognostic values of risk model for evaluating 1‐year, 3‐year and 5‐year survival were 0.82, 0.74 and 0.72 (Figure 6D). Moreover, higher level of *APOA5*, *CXCL8* and *G6PD* was observed in high‐risk HCC tissues, while level of *ADH4*, *CFHR3*, *FTCD* and *PON1* was reduced (Figure 6D). Furthermore, we enrolled the information of age, sex, BMI, T stage, N stage, M stage, tumour stage, tumour grade and risk score for univariate and multivariate Cox regression analysis in HCC individuals in TCGA. It was demonstrated that the risk score was an independent biomarker to evaluate OS in HCC patients in TCGA (Figure 6F,G). We then analysed the prognostic value in HCC tissues from test cohort ICGC. Following classifying HCC patients of ICGC into the two groups (Figure 7A), higher proportion of death cases (Figure 7B) and lower OS time (Figure 7C; HR = 2.86, CI = 1.52–5.26) were observed in the high‐risk group. The prognostic value of risk model in HCC tissues in ICGC for evaluating 1‐year and 3‐year survival were 0.81 and 0.70 (Figure 7D). Expression of *APOA5*, *CXCL8* and *G6PD* was also elevated in HCC tissues from ICGC, whereas expression of *ADH4*, *CFHR3*, *FTCD* and *PON1* was decreased (Figure 7E). Similarly, enrolling the information of sex, age, tumour stage and risk score into univariate and multivariate Cox regression analysis, risk score both had significance (Figure 7F,G). All these evidences exhibited that the risk model established by the significant IAF genes can act as prognostic biomarkers for HCC tissues.

### 2.22 The risk model reflected the immune characteristic and helped to predict the respond of ICI in HCC

**FIGURE 8:**
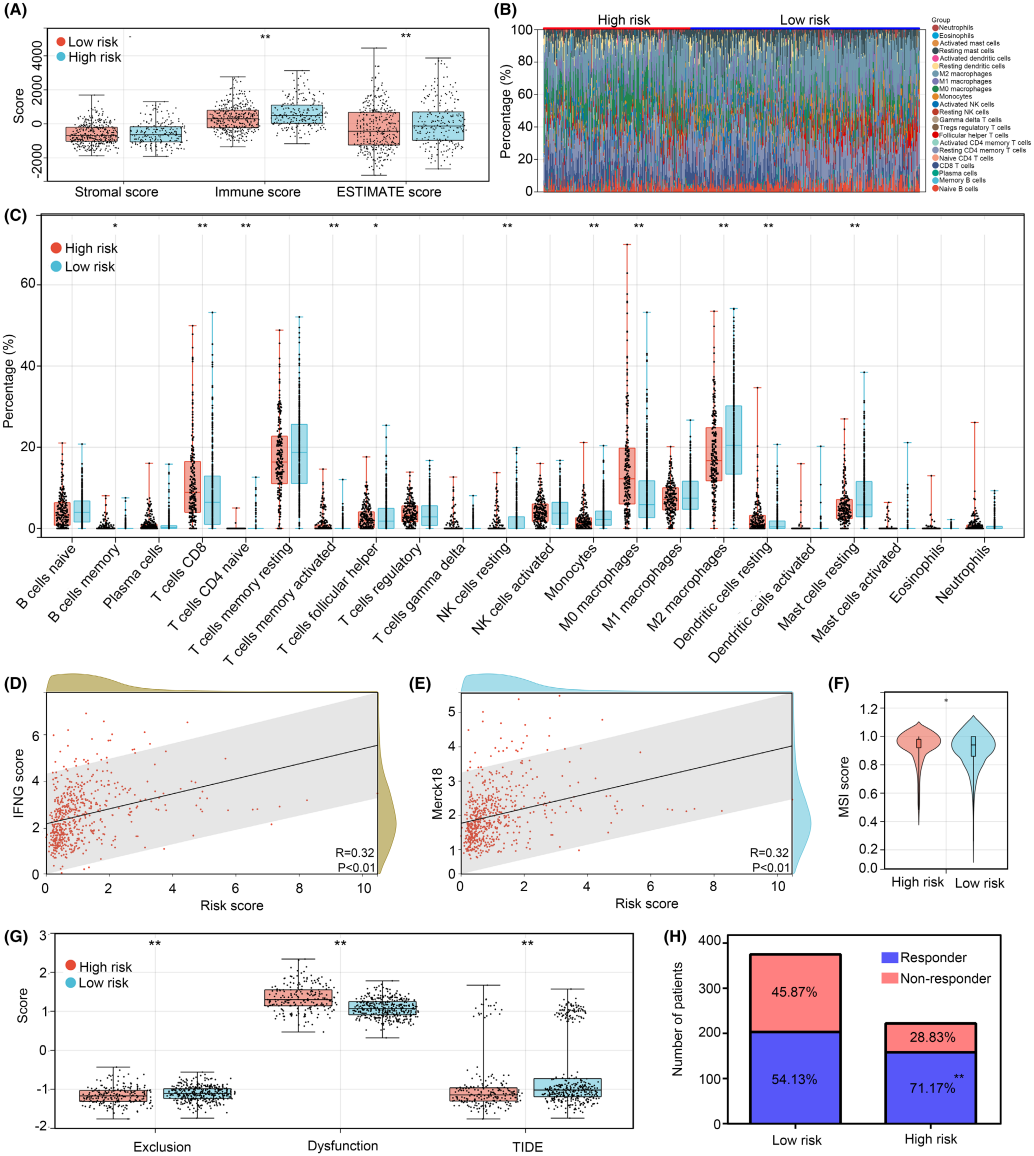
The risk model can reflect the immune characteristic and help to predict the respond of ICI in HCC. (A) Stomal score, immune score and ESTIMATE score between the high‐ and low‐risk groups. (B) Cibersort was used to translate the gene expression matrix of HCC tissues into expression level of 22 immune cells. (C) Differential levels of 22 immune cells between the high‐ and low‐risk groups. (D) The co‐relationship between risk score and IFNG score. (E) The co‐relationship between risk score and Merck18 score. (F) MSI score between the high‐ and low‐risk groups. (G) Exclusion score, dysregulation score and TIDE score between HCC tissues in the high‐ and low‐risk score. (H) The responder rate was predicted in HCC tissues between the high‐ and low‐risk groups. *, *p* < 0.05; **, *p* < 0.01.

As inflammation and ferroptosis crosstalk with immune environment, we then analysed whether the risk model can reflect the immune characteristic in HCC. HCC tissues from train cohort (TCGA) and test cohort (ICGC) was merged, and ESTIMATE algorithm was performed to measure immune parameters in HCC tissues in the high‐ and low‐risk groups. Elevated immune score and ESTIMATE score were observed in the HCC tissues in the high‐risk group (Figure 8A). Moreover, expression levels of 22 immune cells were calculated by Cibersort (Figure 8B). Higher levels of CD8 T cell, naïve CD4 T cell, activated memory T cell, M0 macrophages and resting dendritic cell were observed in HCC tissues in the high‐risk group, while the level of follicular helper T cell, resting NK cell, monocytes, M2 macrophages and resting mast cells were reduced (Figure 8C). These evidences indicated that HCC tissues in the high‐risk group tend to be ‘hot tumours’, and risk model may have the function of identifying ‘hot tumours’. Therefore, we then analysed whether risk model can help to predict the respond of ICI treatment in HCC. TIDE analysis was performed, risk score was positively related to IFNG score (*R* = 0.32, *p* < 0.01; Figure 8D) and Merck18 (*R* = 0.32, *p* < 0.01; Figure 8E). Moreover, through TIDE analysis, MSI score was found higher in HCC tissues in the high‐risk group compared with those in the low‐risk group (Figure 8F). Elevated dysregulation score combined with reduced exclusion and TIDE score were discovered in HCC tissues with high risk (Figure 8G). Furthermore, HCC tissues in the high‐risk group was predicted to had higher respond rate in ICI treatment (Figure 8H). These evidences indicated that the risk model may had function to predict ‘hot tumour’ and the respond of ICI treatment.

### 2.23 The risk model served as diagnostic biomarker for distinguish adjacent tissues and HCC tissues

**FIGURE 9:**
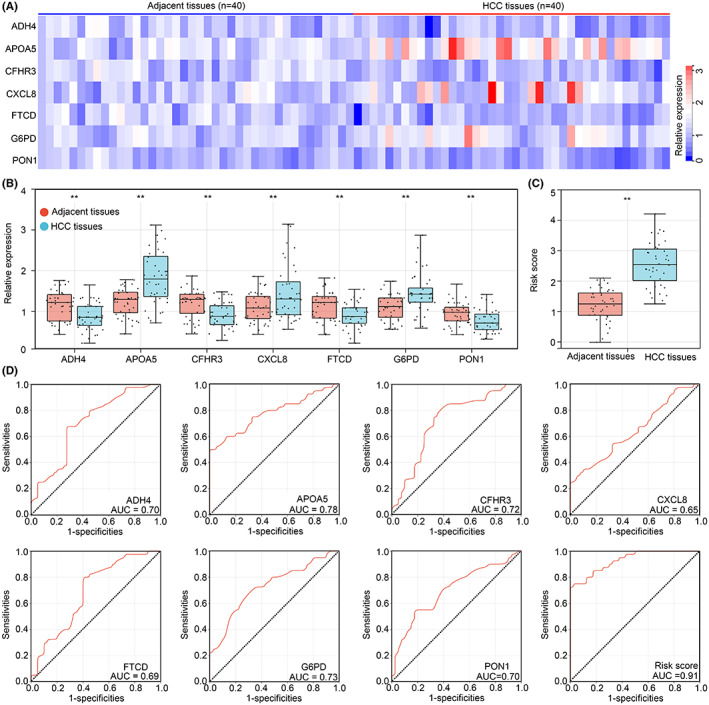
The risk model can act as diagnostic biomarker for distinguish adjacent tissues and HCC tissues. (A) qRT‐PCR was used to detect the expression of *ADH4*, *APOA5*, *CFHR3*, *CXCL8*, *FTCD*, *G6PD* and *PON1* in HCC tissues (*n* = 40) and adjacent tissues (*n* = 40) from our research group. (B) The expression differences of *ADH4*, *APOA5*, *CFHR3*, *CXCL8*, *FTCD*, *G6PD* and *PON1* between HCC tissues (*n* = 40) and adjacent tissues (*n* = 40) from our research group. (C) The expression of *ADH4*, *APOA5*, *CFHR3*, *CXCL8*, *FTCD*, *G6PD* and *PON1* in per tissues were substituted into the formula (risk model), and higher risk score was found in HCC tissues compared with adjacent tissues. (D) The diagnostic value of *ADH4*, *APOA5*, *CFHR3*, *CXCL8*, *FTCD*, *G6PD*, *PON1* and risk score for distinguishing HCC tissues and adjacent tissues. **, *p* < 0.01.

We verified the practicability of risk model in 40 pair HCC tissues and adjacent tissues from our research group. Levels of the seven IAF genes including *ADH4*, *APOA5*, *CFHR3*, *CXCL8*, *FTCD*, *G6PD* and *PON1* in each HCC tissue and adjacent tissue were checked by qRT‐PCR (Figure 9A). Levels of *ADH4*, *CFHR3*, *FTCD* and *PON1* were decreased in the HCC tissues compared with adjacent tissues, and *APOA5*, *CXCL8* and *G6PD* expression was elevated (Figure 9B). We substituted the expression of these genes into the formula (risk model). Interestingly, the risk score was also highly expressed in HCC tissues (Figure 9C). Followed by performing ROC analysis, we exhibited that the diagnostic value of risk score (AUC = 0.91) was higher than each single gene including *ADH4* (AUC = 0.70), *APOA5* (AUC = 0.78), *CFHR3* (AUC = 0.72), *CXCL8* (AUC = 0.65), *FTCD* (AUC = 0.69), *G6PD* (AUC = 0.73) and *PON1* (AUC = 0.70) to distinguish HCC tissues and adjacent tissues. These evidences exhibited that the risk model may be a remarkable tool for HCC diagnosis.

### 2.24 The risk model helped for optimising the choice of immunotherapy

**FIGURE 10:**
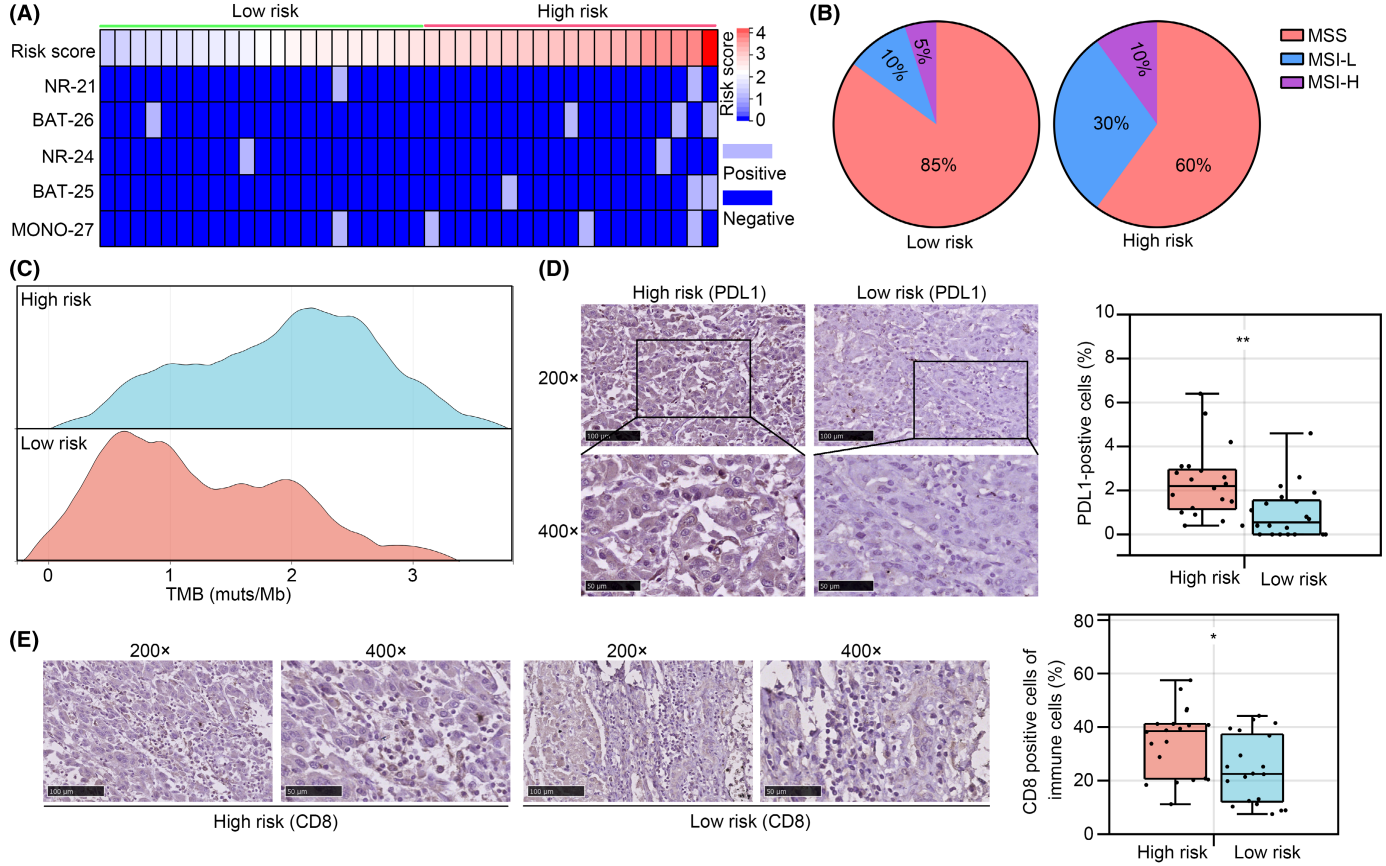
The risk model can help to optimize the choice of immunotherapy. (A) NR‐21, BAT‐26, NR‐24, BAT‐25 and MONO‐27 site mutation condition in HCC tissues from our research group. (B) Different distribution of MSS, MSI‐L and MSI‐H in the high‐and low‐risk group. (C) The difference of TMB between HCC tissues in the high‐ and low‐risk groups. (D) The expression of PDL1 in HCC tissues in the high‐ and low‐risk groups. (E) The positive rate of CD8 in immune cells in HCC tissues in the high‐ and low‐risk groups. *, *p* < 0.05; **, *p* < 0.01.

We then reviewed the clinical characters of patients with HCC tissues (*n* = 40) from our research group, and tried to find the correlationship between the risk model calculating by substituting the expression of genes and patient clinical characters. Among the patients, total 2, 4, 2, 3 and 4 patients had NR‐21, BAT‐26, NR‐24, BAT‐25 and MONO‐27 site mutation (Figure 10A). We divided our HCC patients into high‐ and low‐risk groups based on medium of risk score, and we found that the patients with MSI‐H and MSI‐L was significantly more in the high‐risk group in comparison with the low‐risk group (Figure 10B). Similarly, we found that patients from our research group in the high‐risk group had higher TMB in comparison to the low‐risk group. Furthermore, through performing IHC, higher PDL1 expression and more immune cells with CD8 expression were found in the HCC tissues having high risk (Figure 10C,D). As higher MSI, TMB, PDL1 and more CD8 cells were biomarkers for respond of ICI,^16^ we considered that risk model can help us to distinguish patients may respond for ICI, thus optimising the choice of immunotherapy.

### 2.25 APOA5 was a novel target for inhibiting HCC via upregulating inflammation and ferroptosis

**FIGURE 11:**
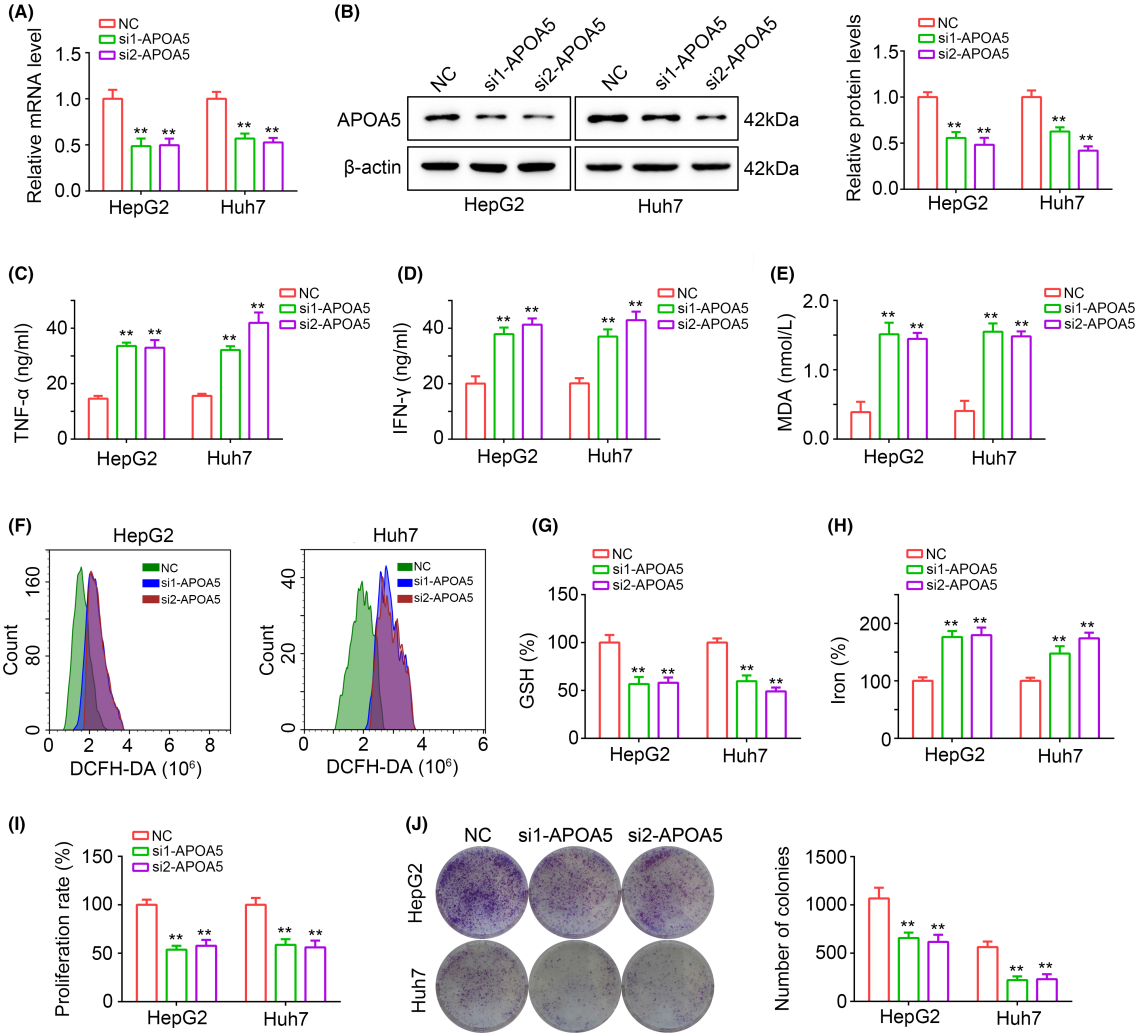
Knockdown of APOA5 can significant reduce the proliferation and colony formation combining with elevating inflammation and ferroptosis levels. (A) RT‐qPCR was used to detect the mRNA level of APOA5 in HepG2 and Huh7 cells after APOA5 knockdown. (B) Western blotting was used to detect the protein level of APOA5 in HepG2 and Huh7 cells after APOA5 knockdown. (C, D) Elisa was used to detect the levels of TNF‐α and IFN‐γ in HepG2 and Huh7 cells after APOA5 knockdown. Levels of MDA (E), ROS (F), GSH (G) and iron (H) were measured in HepG2 and Huh7 cells after APOA5 knockdown. (I) CCK‐8 was used to detect the proliferation rate of HepG2 and Huh7 cells after APOA5 suppression. (J) Colony formation assay was used to detect the colony formation ability of HepG2 and Huh7 cells after APOA5 suppression. **, *p* < 0.01.

Moreover, after reviewing previous studies, we found that, as a gene in lipid regulation,^17^ the effects of APOA5 on HCC progression, inflammation and ferroptosis has not been reported in previous studies, thus catching our attention. Utilising two siRNAs, APOA5 knockdown cells were constructed (Figure 11A,B). Through performing ELISA, we found that HepG2 and Huh7 with APOA5 knockdown had elevating TNF‐α (Figure 11C) and IFN‐γ levels (Figure 11D). Then, we detected the levels of MDA, a product of lipid peroxidation in HCC cells, and found that suppression of APOA5 in HCC cells increased the MDA levels in HepG2 and Huh7 cells (Figure 11E). Moreover, we found that ROS levels (Figure 11F) were significantly increased in HepG2 and Huh7 cells with APOA5 knockdown, while the GSH (Figure 11G) in cells were reduced. Moreover, we found that the iron levels in HCC cells were increased (Figure 11H). These results indicated that knockdown of APOA5 may increase inflammation and ferroptosis. Furthermore, we found that knockdown of APOA5 reduced cell proliferation (Figure 1I) and colony formation (Figure 1J). Therefore, we considered that APOA5 was a novel target for inhibiting HCC via upregulating inflammation and ferroptosis.

## 3 DISCUSSION

Recently, immunotherapy, radio‐chemotherapy and targeting therapy were used jointly to suppress HCC progression and elevate the prognosis.^18^ HCC was highly heterogeneous, and the degree of inflammation and immune cell infiltration in its microenvironment affect the efficacy of treatment of HCC, especially immunotherapy. However, due to low MSI and immune suppressive microenvironment, patients with HCC commonly had low respond rate of immunotherapy including ICI.^19^

More and more recognized that inflammation plays as ‘double‐edged sword’ in HCC progression. On the one hand, inflammation in tumour tissue can induce inflammation‐related death of tumour cells, such as ferroptosis, thus suppressing the progression of HCC. Similarly, previous studies indicated that, during the radio‐therapy, chemotherapy and immunotherapy, immune cells including CD8 T cells and NK cells are activated, and secrete a series of inflammatory factors including TNF‐α and INF‐γ to induce inflammation‐related death of tumour cells.^20^ However, chronic inflammation accelerates the progression of tumour via activating a series of inflammation relative pathways, including NF‐kappaB and JAKs‐STATs.^21^ Moreover, tumour cells would change the inflammatory microenvironment to form properties of immunosuppression. Inflammation‐induced ferroptosis is favourable for cancer therapy, while the products of cells undergoing ferroptosis can act as immunogen to induce immune cell infiltration.^22^ Previous studies indicated that ICI based on the data obtaining from high‐throughput sequencing is helpful for the precise treatment of HCC.^23^, ^24^ Herein, therefore, we first aimed to explore novel biomarker associated with inflammation and ferroptosis in HCC tissues, and its effect on guiding immunotherapy via analysing high‐throughput sequencing data.

In the current study, analysis of the gene expression profile of HCC tissues in TCGA, total 224 IAF genes were identified. Then, through selection of lasso Cox, univariate Cox regression analysis and multivariate Cox regression analysis, seven IAF genes including *ADH4*, *APOA5*, *CFHR3*, *CXCL8*, *FTCD*, *G6PD* and *PON1* were used for construction of a risk model. Following checking in train corhort (TCGA) and test cohort (ICGC), risk model was found to have potential to play as an independent biomarker for prognostic evaluation in HCC. Previous studies indicated that ‘hot tumour’ with more immune cell infiltration, especially T cell infiltration, had higher responder rate for ICI treatment.^25^ Interesting, the high‐risk group classifying by the risk model was found to had more immune score and higher levels of CD8 T cell, naïve CD4 T cell, activated memory T cell, which were predicted to have higher respond rate to ICI. These results indicated that the risk model may help to identify ‘hot tumour’, and had potential to guide the ICI choice.

*ADH4‐*encoded protein belong to the family of alcohol dehydrogenase. In a physiological state, it oxidizes long chain omega‐hydroxy fatty acids into 20‐oxoarachidonate, and induces the reduction of benzoquinones.^26^ *APOA5*‐encoded protein is an apolipoprotein, involved in regulating the plasma triglyceride levels.^27^ *APOA5‐*encoded protein is a component of high‐density lipoprotein.^28^ *CFHR3*‐encoded protein is a secreted protein, which belongs to the complement factor H‐related protein family. *CFHR3‐*encoded protein is a key factor for complement‐regulated inflammatory responses.^29^ *CXCL8‐*encoded protein is also named as interleukin‐8 (IL‐8), and is a major mediator of the inflammatory response.^30^ *FTCD*‐encoded protein exhibits both transferase and deaminase activity, induces channels 1‐carbon units from formiminoglutamate to the folate pool.^31^ *G6PD*‐encoded protein is the rate‐limiting step of the oxidative pentose‐phosphate pathway, via providing reducing power (NADPH) and pentose phosphates for fatty acid and nucleic acid synthesis.^32^ *PON1‐*encoded protein belongs to the paraoxonase family, possessing lactonase and ester hydrolase activity.^33^ The protein is secreted by the kidney and liver, and has potential to bind with high‐density lipoprotein, regulating the process of lipid synthesis.^34^ Dysregulation of these genes had been observed in atherosis, autoimmune disease and tumours, including HCC. For example, *ADH4* level was reduced in HCC, and low level of *ADH4* was related to poor prognosis.^35^ Elevated *G6PD* was observed in HCC tissues, while it promotes the progression of HCC via suppressing ferroptosis.^36^ Similarly, our previous study indicated that *CFHR3* was respond to hypoxia, and reduced in HCC with hypoxic microenvironment.^37^ Consistent with previous studies, through qRT‐PCR, *ADH4*, *CFHR3*, *FTCD* and *PON1* levels were found to reduce in the HCC tissues compared with adjacent tissues, and *APOA5*, *CXCL8* and *G6PD* expression was elevated. It is worth noting that the risk model constructed by them exhibited higher diagnostic value than each single genes. This may be one of shining points of the point. Moreover, we found that APOA5 was a novel target in HCC. Knockdown of APOA5 can significant reduce the proliferation and colony formation combining with elevating inflammation and ferroptosis levels.

Previous studies that patients with higher MSI and TMB commonly respond for ICI, and they were set as biomarkers to decide whether to proceed ICI.^38^ Therefore, we plugged in the expression of each gene into the formula (risk model), and classifying the patients from our research group into the high‐ and low‐risk groups. Through reviewing clinical characteristic, it was indicated that HCC individuals in the high‐risk group had higher MSI and TMB. Finally, we suggested that tissues in the high‐risk group had higher PDL1 expression and immune cells with CD8 using IHC. These results were consistent with above‐mentioned bioinformatics analysis, verifying that the risk model can distinguish ‘hot tumour’. Therefore, we consider the risk model had potential to optimize the choice of ICI in patients with HCC by reflecting the immune characteristics in HCC tissues.

## 4 CONCLUSIONS

In conclusion, we considered APOA5 was a novel target for suppressing HCC via simultaneously elevating inflammation and ferroptosis levels, and the signature constructed by seven IAF genes including *ADH4*, *APOA5*, *CFHR3*, *CXCL8*, *FTCD*, *G6PD* and *PON1* can act as a biomarker for optimising the diagnosis, prognosis evaluation and immunotherapy options in patients with HCC.

## AUTHOR CONTRIBUTIONS

**Wan‐Yuan Ruan:** Data curation (lead); methodology (lead). **Lu Zhang:** Data curation (equal); methodology (lead). **Shan Lei:** Formal analysis (equal); investigation (supporting); methodology (equal). **Zhi‐Rui Zeng:** Data curation (supporting); formal analysis (supporting); methodology (equal); writing – original draft (lead); writing – review and editing (lead). **Yu‐Shi Yang:** Data curation (supporting); resources (supporting); validation (supporting); visualization (equal). **Wen‐Peng Cao:** Data curation (equal); resources (equal); software (equal). **Qiu‐Yao Hao:** Investigation (supporting); resources (supporting); software (supporting). **Min Lu:** Conceptualization (lead). **Xiao‐Bin Tian:** Conceptualization (equal); funding acquisition (lead); project administration (lead); supervision (equal). **Pai‐Lan Peng:** Conceptualization (equal); funding acquisition (lead); project administration (lead); supervision (lead).

## FUNDING INFORMATION

The study was supported by National Natural Science Foundation of China (No. 82060541), Project of Guizhou Science and Technology Department [No. Qiankehe support (2021) general 089], National nature Cultivation Project of Affiliated Hospital of Guizhou Medical University [No. gyfynsfc(2020)‐4], Doctoral research start‐up project of Affiliated Hospital of Guizhou Medical University (No. gyfybsky‐2021‐25) and Project of Health Commission of Guizhou Province (No. gzwkj2023‐170).

## CONFLICT OF INTEREST STATEMENT

The authors declare no conflicts of interest.

## Supporting information


Table S1.

Table S2.

## Data Availability

The data used to support the findings of this study are available from the corresponding author upon reasonable request.

## References

[1] Chakraborty E , Sarkar D . Emerging therapies for hepatocellular carcinoma (HCC). Cancers. 2022;14(11):2798. 35681776 10.3390/cancers14112798 PMC9179883

[2] Sugawara Y , Hibi T . Surgical treatment of hepatocellular carcinoma. Biosci Trends. 2021;15(3):138‐141. 33746184 10.5582/bst.2021.01094

[3] Bao MH , Wong CC . Hypoxia, metabolic reprogramming, and drug resistance in liver cancer. Cell. 2021;10(7):1715. 10.3390/cells10071715 PMC8304710 34359884

[4] Chen S , Cao Q , Wen W , Wang H . Targeted therapy for hepatocellular carcinoma: challenges and opportunities. Cancer Lett. 2019;460:1‐9. 31207320 10.1016/j.canlet.2019.114428

[5] Sangro B , Sarobe P , Hervás‐Stubbs S , Melero I . Advances in immunotherapy for hepatocellular carcinoma. Nat Rev Gastroenterol Hepatol. 2021;18(8):525‐543. 33850328 10.1038/s41575-021-00438-0 PMC8042636

[6] Lleo A , Rimassa L , Colombo M . Hepatotoxicity of immune check point inhibitors: approach and management. Dig Liver Dis. 2019;51(8):1074‐1078. 31296449 10.1016/j.dld.2019.06.017

[7] Singh N , Baby D , Rajguru JP , Patil PB , Thakkannavar SS , Pujari VB . Inflammation and cancer. Ann Afr Med. 2019;18(3):121‐126. 31417011 10.4103/aam.aam_56_18 PMC6704802

[8] Mantovani A , Allavena P , Sica A , Balkwill F . Cancer‐related inflammation. Nature. 2008;454(7203):436‐444. 18650914 10.1038/nature07205

[9] Hou J , Karin M , Sun B . Targeting cancer‐promoting inflammation – have anti‐inflammatory therapies come of age? Nat Rev Clin Oncol. 2021;18(5):261‐279. 33469195 10.1038/s41571-020-00459-9 PMC8978805

[10] Hsu SK , Li CY , Lin IL , et al. Inflammation‐related pyroptosis, a novel programmed cell death pathway, and its crosstalk with immune therapy in cancer treatment. Theranostics. 2021;11(18):8813‐8835. 34522213 10.7150/thno.62521 PMC8419056

[11] Yan HF , Zou T , Tuo QZ , et al. Ferroptosis: mechanisms and links with diseases. Signal Transduct Target Ther. 2021;6(1):49. 33536413 10.1038/s41392-020-00428-9 PMC7858612

[12] Lei G , Zhuang L , Gan B . Targeting ferroptosis as a vulnerability in cancer. Nat Rev Cancer. 2022;22(7):381‐396. 35338310 10.1038/s41568-022-00459-0 PMC10243716

[13] Zhao L , Zhou X , Xie F , et al. Ferroptosis in cancer and cancer immunotherapy. Cancer Commun. 2022;42(2):88‐116. 10.1002/cac2.12250 PMC8822596 35133083

[14] Chen X , Kang R , Kroemer G , Tang D . Ferroptosis in infection, inflammation, and immunity. J Exp Med. 2021;218(6):e20210518. 33978684 10.1084/jem.20210518 PMC8126980

[15] Danaher P , Warren S , Lu R , et al. Pan‐cancer adaptive immune resistance as defined by the tumor inflammation signature (TIS): results from the cancer genome atlas (TCGA). J Immunother Cancer. 2018;6(1):63. 29929551 10.1186/s40425-018-0367-1 PMC6013904

[16] Bagchi S , Yuan R , Engleman EG . Immune checkpoint inhibitors for the treatment of cancer: clinical impact and mechanisms of response and resistance. Annu Rev Pathol. 2021;16:223‐249. 33197221 10.1146/annurev-pathol-042020-042741

[17] Garelnabi M , Lor K , Jin J , Chai F , Santanam N . The paradox of ApoA5 modulation of triglycerides: evidence from clinical and basic research. Clin Biochem. 2013;46(1–2):12‐19. 23000317 10.1016/j.clinbiochem.2012.09.007 PMC3534811

[18] Cheng AL , Hsu C , Chan SL , Choo SP , Kudo M . Challenges of combination therapy with immune checkpoint inhibitors for hepatocellular carcinoma. J Hepatol. 2020;72(2):307‐319. 31954494 10.1016/j.jhep.2019.09.025

[19] Giraud J , Chalopin D , Blanc JF , Saleh M . Hepatocellular carcinoma immune landscape and the potential of immunotherapies. Front Immunol. 2021;12:655697. 33815418 10.3389/fimmu.2021.655697 PMC8012774

[20] Gemelli M , Noonan DM , Carlini V , et al. Overcoming resistance to checkpoint inhibitors: natural killer cells in non‐small cell lung cancer. Front Oncol. 2022;12:886440. 35712510 10.3389/fonc.2022.886440 PMC9194506

[21] Fan Y , Mao R , Yang J . NF‐κB and STAT3 signaling pathways collaboratively link inflammation to cancer. Protein Cell. 2013;4(3):176‐185. 23483479 10.1007/s13238-013-2084-3 PMC4875500

[22] Wang F , He J , Xing R , Sha T , Sun B . Molecular mechanisms of ferroptosis and their role in inflammation. Int Rev Immunol. 2023;42(1):71‐81. 34918993 10.1080/08830185.2021.2016739

[23] Li M , Kaili D , Shi L . Biomarkers for response to immune checkpoint inhibitors in gastrointestinal cancers. World J Gastrointest Oncol. 2022;14(1):19‐37. 35116101 10.4251/wjgo.v14.i1.19 PMC8790411

[24] Jia Q , Chu H , Jin Z , Long H , Zhu B . High‐throughput single‐сell sequencing in cancer research. Signal Transduct Target Ther. 2022;7(1):145. 10.1038/s41392-022-00990-4 PMC9065032 35504878

[25] Galon J , Bruni D . Approaches to treat immune hot, altered and cold tumours with combination immunotherapies. Nat Rev Drug Discov. 2019;18(3):197‐218. 30610226 10.1038/s41573-018-0007-y

[26] Yin SJ , Chou CF , Lai CL , Lee SL , Han CL . Human class IV alcohol dehydrogenase: kinetic mechanism, functional roles and medical relevance. Chem Biol Interact. 2003;143‐144:219‐227. 10.1016/s0009-2797(02)00167-9 12604207

[27] Guardiola M , Ribalta J . Update on APOA5 genetics: toward a better understanding of its physiological impact. Curr Atheroscler Rep. 2017;19(7):30. 28500476 10.1007/s11883-017-0665-y

[28] Su X , Peng D . The exchangeable apolipoproteins in lipid metabolism and obesity. Clin Chim Acta. 2020;503:128‐135. 31981585 10.1016/j.cca.2020.01.015

[29] Józsi M , Tortajada A , Uzonyi B , Goicoechea de Jorge E , Rodríguez de Córdoba S . Factor H‐related proteins determine complement‐activating surfaces. Trends Immunol. 2015;36(6):374‐384. 25979655 10.1016/j.it.2015.04.008

[30] Asokan S , Bandapalli OR . CXCL8 signaling in the tumor microenvironment. Adv Exp Med Biol. 2021;1302:25‐39. 34286439 10.1007/978-3-030-62658-7_3

[31] Zhang W , Wu C , Ni R , Yang Q , Luo L , He J . Formimidoyltransferase cyclodeaminase prevents the starvation‐induced liver hepatomegaly and dysfunction through downregulating mTORC1. PLoS Genet. 2021;17(12):e1009980. 34941873 10.1371/journal.pgen.1009980 PMC8741050

[32] Yang HC , Wu YH , Yen WC , et al. The redox role of G6PD in cell growth, cell death, and cancer. Cell. 2019;8(9):1055. 10.3390/cells8091055 PMC6770671 31500396

[33] Lou‐Bonafonte JM , Gabás‐Rivera C , Navarro MA , Osada J . PON1 and Mediterranean diet. Nutrients. 2015;7(6):4068‐4092. 26024295 10.3390/nu7064068 PMC4488773

[34] Bacchetti T , Ferretti G , Carbone F , et al. Dysfunctional high‐density lipoprotein: the role of myeloperoxidase and paraoxonase‐1. Curr Med Chem. 2021;28(14):2842‐2850. 32674726 10.2174/0929867327999200716112353

[35] Wei RR , Zhang MY , Rao HL , Pu HY , Zhang HZ , Wang HY . Identification of ADH4 as a novel and potential prognostic marker in hepatocellular carcinoma. Med Oncol. 2012;29(4):2737‐2743. 22147505 10.1007/s12032-011-0126-3

[36] Cao F , Luo A , Yang C . G6PD inhibits ferroptosis in hepatocellular carcinoma by targeting cytochrome P450 oxidoreductase. Cell Signal. 2021;87:110098. 34325001 10.1016/j.cellsig.2021.110098

[37] Zeng Z , Lei S , Wang J , et al. A novel hypoxia‐driven gene signature that can predict the prognosis of hepatocellular carcinoma. Bioengineered. 2022;13(5):12193‐12210. 35549979 10.1080/21655979.2022.2073943 PMC9276011

[38] Wang Y , Tong Z , Zhang W , et al. FDA‐approved and emerging next generation predictive biomarkers for immune checkpoint inhibitors in cancer patients. Front Oncol. 2021;11:683419. 34164344 10.3389/fonc.2021.683419 PMC8216110

